# The views and experiences of general dental practitioners (GDP’s) in West Yorkshire who used the International Caries Detection and Assessment System (ICDAS) in research

**DOI:** 10.1371/journal.pone.0223376

**Published:** 2019-10-04

**Authors:** Maria Ishaq Khattak, Julia Csikar, Karen Vinall, Gail Douglas

**Affiliations:** 1 School of Dentistry, University of Leeds, Leeds, United Kingdom; 2 Institute of Public Health & Social Sciences, Khyber Medical University, Khyber Pakhtunkhwa, Peshawar, Pakistan; Northumbria University, UNITED KINGDOM

## Abstract

**Objective:**

To explore, through face to face interviews with a selection of General Dental Practitioners (GDPs), their views and experiences of having used the International Caries Detection and Assessment System (ICDAS) within primary care research studies for recording caries.

**Methods:**

This qualitative study involved one on one interviews with eight GDP’s who had previously used ICDAS on patients in their dental practices as part of a research study. The participants were selected from among those who had taken part in two clinical studies in the UK using convenient, but purposive sampling. The interviews were tape-recorded and transcribed; the data analysis was conducted by thematic analysis.

**Results:**

GDP’s indicated their beliefs that ICDAS had an important role in caries prevention but reported four main barriers while using the full (6 caries stages) ICDAS coding system in their practices: lack of simplicity of coding, financial implications and time consumption (in both training and use of ICDAS) and inadequate undergraduate training. An overarching theme identified from the GDPS was the willingness to offer potential solutions to their barriers which might improve the utilisation of the system in primary care.

**Conclusion:**

The GDPs experienced common obstacles in using ICDAS in the primary care setting, many of which have relatively straight-forward solutions which they put forward themselves such as: incentivisation, undergraduate-level training in ICDAS for both dentists and nurses and computerized data entry. Further qualitative and quantitative research is needed on how to facilitate the utilisation of the system in dental practice. It is also recommended to explore the influences of wider agencies on influencing primary dental care professionals’ caries management, including appropriate recording of diagnosis and risk assessment.

## Introduction

Dental caries is a multifaceted disease [1]. In 2010, it was estimated that untreated caries in deciduous teeth affects 9% of the population globally which equates to 621 million people worldwide [2]. For some time there has been no single standard system for caries detection and/or assessment that scientists and general dentists agree upon collectively [3] with evidence of 29 different detection systems being used in clinical practice and research around the world for the description and diagnosis of the caries process [4]. It was also suggested that many of these detection systems were insufficient due to poor sensitivity [5]. The International caries detection and assessment system (ICDAS) was developed to address disharmonies of the previous systems by using their best elements and demonstrating abilities in standardising caries assessment [6]. This system combines components of various caries classification systems into one standard system by the use of a six-point scale that ranges from the earliest visible stage of enamel caries to extensive lesions with cavitation exposing dentine in order to describe caries severity stages (Table 1) [7].

**Table 1:** Criteria for the full ICDAS scoring system (International Caries Detection and Assessment System coordinating committee 2005b).

Score	ICDAS criteria
0	Sound
1	First visual change in enamel (seen only after prolonged air drying or restricted to within the confines of a pit or fissure)
2	Distinct visual change in enamel
3	Localized enamel breakdown (without clinical visual signs of dentinal involvement)
4	Underlying dark shadow from dentine
5	Distinct cavity with visible dentine
6	Extensive distinct cavity with visible dentine

The ICDAS system was developed by dental clinicians and academics from various dental institutes across Europe and America [8]. This development led to a recommendation within the European Core Curriculum in cariology, that the ICDAS system should be advocated in undergraduate teachings [9]. The World Health Organization (WHO) has utilised the Decay Missing Filled (DMF) index in oral health assessment surveys such as the national dental surveys in the UK carried out every decade since 1968, the DMF index has been used in several surveys in Asia, Africa, North America, Europe and Australia [10,11]. The ICDAS system has been used by researchers for surveys of dental caries experience also, but less widely [12,13].

The ICDAS system has been used in research for about a decade, which is a promising indicator of the adoption of the method in the field of research [14]. The ICDAS system has been effective when measured against established methods for caries detection and is used effectively in research [15]. When the ICDAS system has been tested against other systems such as the WHO index and laser fluorescence; it was found that the ICDAS system was more effective than some other systems in the early detection of caries [16,17].

Current literature suggests that GDP’s and undergraduate dental students till date are being taught to use the conventional DMF index for assessment of dental caries which was developed by Klein, Palmer and Knuston in 1938 [18]. One of the most essential activities of general dental practice is decision making. A wide variety of decision making processes amongst dentists have been reported [19]. The process of decision is not straightforward; it is influenced by various features such as learnt norms, years of qualification, years in practice, public or private settings [20]. The ICDAS system when used in dental clinics intends to deliver high quality information about caries activity and when combined with caries risk assessment it facilitates a personalised care pathway for every patient [21]. ICDAS is a meticulous caries assessment system that allows for early identification of caries activity, it encourages practitioners mind to think in a preventive way from the start [22].

Although some reports suggests that there are a number of dental undergraduate establishments using the ICDAS system for teaching purposes [23], there is weak evidence about adoption of the ICDAS system in clinical practice, since it is a newer system compared to the conventional WHO DMF index, it is currently unknown if ICDAS does change clinical practice [24]. There is a lack of evidence as to how many dentists are trained to use the system and how many actually use it in their dental practices. Utilisation and adaption of the ICDAS system by GDP’s and for oral health assessment survey remains less well researched.

Research to date has focussed on quantitative methodologies to establish the reliability, feasibility, validity, reproducibility and practicality of using ICDAS. However, to our knowledge none of the studies have looked at the GDP’s perceptions about barriers and facilitating factors while using the ICDAS index in research or clinical settings. There is a gap in the literature regarding what factors exist that may hinder or help practitioners to use the ICDAS index in clinical practice. To address this gap, a qualitative one on one interview based study was conducted in the United Kingdom to explore the views and experiences of GDP’s and to determine the factors responsible for increasing the uptake of the ICDAS system in the future.

## Methods

### Participants

Twelve GDP’s who had previously taken part in research projects using ICDAS were approached via email and telephone to participate in this research using convenience but purposive sampling methods. Ethical approval was given by the University of Leeds School of Dentistry Research Ethics Committee (DREC Reference: 050416/MK/196).

The GDPs were contacted by a member of the research team that they originally worked with (KV) and given sufficient information regarding the aim and rationale to allow them to consider if they would like to take part in the proposed study. This way the details of GDP’s who had taken part in previous studies were not shared with the interviewer (MIK), protecting their personal details.

The research experiences of the GDPs varied slightly depending on which previous research study they had participated in. One was a trial involving mixed dentition children while the other was of adults. The training that each received was very similar and from the same ICDAS-experienced clinician. This comprised of face to face didactic photograph-based training after self-directed preparatory training using a 90-minute e-learning package [25], face to face training continued until participants were confident and correctly identified codes from photographic images reliably. Their training varied only with respect to the dental charts used for mixed or permanent dentition.

Those wanting further information about the proposed study were put in touch with the interviewer via a brief telephone call, and the interested participants received an email copy of the study information leaflet.

### Procedure and interview schedule

In order to explore in depth the insight and experiences of the GDP’s on the ICDAS system, a one on one interview method was chosen for collecting data. One to one interviews were conducted using a topic guide that was developed based on the literature review and was piloted with first interviewee by the researcher (MIK) along with an experienced qualitative researcher (KV), to ensure the questions were clear and facilitated discussion, the data from the pilot was utilised within the study. A total of 8 GDP’s willing to participate were recruited and consented at a personal meeting prior to undertaking the interview on the same day. Those 8 general dental practitioners comprised of four males and four females. The interview participants consisted of 4 general dentists, 2 former general dentists who were waiting to begin their speciality training (maxillofacial surgery, oral medicine) and 2 general dentists that had recently started working in the community dental setting.

To minimize the burden of participation, the length of the interviews were restricted to around 30 minutes. The interviews took place in participant’s dental practices with only the researcher and participant present.

The risk of exposing identifiable information about the participants was countered by giving every participant a penname of their choice before beginning the interview and they were assured that there was no data from which their identity could be revealed. Before the interview took place participants were asked for audio-recording permission, to which all agreed.

The participants were given a copy of the ICDAS coding sheet before the interview started, in order to remind them of the coding in case they had not used the ICDAS system recently. They were asked exploratory questions with the purpose of putting them at ease, so that they would be able to express their views and beliefs about the ICDAS system; what it meant to them in the previous research study and in clinical settings.

### Analysis

The audio-recorded sessions were transcribed verbatim with anonymization. The participants were given an opportunity to verify that their views and opinions had been represented in a fair manner, none requested any change or correction.

Interview transcripts and analysis were organized through NVivo 10 software. Phenomenology was selected as a foundation methodology for this study. It is the approach that helps in explaining and identifying the importance of people’s experiences [26]. A pilot interview was performed by the researcher, which was assessed by the supervisory team and feedback was conveyed. Data was analysed using Thematic Analysis, as it is systematic method that helps in identifying, organising, analysing the rich data set and reporting meaningful patterns and is a common method used for problem framing while using phenomenological approach in the data analysis [27,28]. The intention for this sort of data analysis was necessary for this research project as it gave an enhanced understanding of characteristics of potential barriers for the GDP’s while using the ICDAS system; attributes that can be used as facilitators for improving the uptake of the system. Based on Braun and Clarke’s 2013 thematic analysis [27], repeated reading of interview transcripts was conducted, initial coding was established by the researcher (MIK), the initial coding was reviewed by the supervisory team and broad level themes were derived from the data.

## Results

### Overview of the results

Overall, 12 GDP’s were initially approached to take part in the research out of which 8 GDP’s consented to take part. To achieve in-depth information about the research topic participants were chosen with varying years in practice as GDPs, gender, age and current working role, their main characteristics are shown in (Table 2).

**Table 2:** Characteristics of interview participants.

Pen-name	Gender	Age Years	Years in practice as GDP	Current working role
ID 1 Male	Male	55 years	20	General Dental Practitioner
ID 2 Male	Male	46 years	13	General Dental Practitioner
ID 3 Male	Male	38 years	12	Community Dental settings, recently left GDP
ID 1 Female	Female	34 years	7	General Dental Practitioner waiting to start training in Oral Medicine
ID 2 Female	Female	30 years	5	General Dental Practitioner
ID 3 Female	Female	25 years	3	Community Dental settings, recently left GDP
ID 4 Female	Female	35 years	8	General Dental Practitioner waiting to start training in Maxillofacial surgery
ID 4 Male	Male	50 years	18	General Dental Practitioner

The analysis used an inductive approach to identify themes as highlighted in the thematic map S1 Fig. The map shows the interrelationship between the themes and illustrates facilitating factors related to using the ICDAS system. The inter-relationship between themes highlighted by the GDP’s, the major themes are presented in red, broken lines present interrelationships between themes, the direct lines present a direct relationship, the potential facilitators for the major themes are presented in purple. The possible underlying factors responsible for the barriers are presented in brown.

### Barriers-related to using the ICDAS system

Four main themes for this category were recognised from the data: lack of simplicity, financial implications, time consumption, and training.

#### Lack of simplicity

Lack of simplicity was one of the main themes identified in the findings. In the eyes of most users of the ICDAS system, they felt it was not easy to use, maintain using it and understand. Lack of simplicity was found in two contexts: difficulty deciding between different codes and difficulty while charting. One of the participants mentioned the word ‘complexity’ while incorporating the system in research:

“*Probably*, *because of the complexity of charting and then trying to analyse that information*. *So*, *all these numbers written down for the mesial surface*, *occlusal surface*, *for 4a*, *for 4*, *7; it’s probably a little bit too much to sort of analyse it really*. *That’s probably the hardest way”*

(ID 2 Male)

When participants had been trained in the use of ICDAS for their respective clinical studies, the trainer returned to participants’ practices on request until participants demonstrated competence. However, all participants interviewed for this study revealed concerns about not completely understanding the ICDAS codes. There were varying levels of understanding; of how to detect and assess caries. Two participants felt that they were not confident about distinguishing between early codes:

“*…it was between the 1’s and 2’s [both non-cavitated enamel caries] that were difficult*. *Once*, *there is a great big stonking hole it’s pretty obvious and anybody can see that…”*

(ID 4 Female)

“..*I think zero*, *1*, *2 how to differentiate between and sometimes weren’t sure which one is 1 or 2…”*

(ID 2 Male)

The ease of use of a new system can vary from one dentist to another and the GDPs differed in their reported level of understanding of the technique. The ability to learn the ICDAS system because of relative complications was remarked by a participant:

*“I think it was a bit too complicated as a first off to be using it on patients for research and having so many different codes that you have not been used to using*.”

(ID 2 Femalea)

#### Financial implications

Financial disadvantage to the GDP’s was one of the most common themes among the participants’ discussions and was one of the important reasons cited for not adopting the ICDAS system, as it has personal cost implications to them as independent contractors. One participant stated:

“… *Obviously the incentive financially isn’t there to use it really [outside of research]*. *So*, *I think that does have a big*, *big factor…”*

(ID 2 Male)

Three participants revealed that the impetus for using the ICDAS system in their clinical practice to detect and then act on early caries lesions in particular was absent and this was based on the simple rule of cost-benefit analyses:

“*I would say*, *we as a dentist are not paid for prevention and by the end of the day clinical decision-making are influenced by other factors as well*.”

(ID 3 Female)

#### Time consuming

Aside from the implied costs of the ICDAS system taking additional time to complete in clinics, other issues with respect to ‘time’ were raised, such as the amount of time it took the GDP’s to examine the teeth systematically and then chart them using the ICDAS system. This issue was a fundamental problem for them. A participant specified:

“…*It does take longer*, *and sometimes that can be a problem…”*

(ID 2 Male)

Two participants indicated that the challenge with time was not only crucial for them as dentists but also other practice staff were not comfortable with designating additional time for thorough examination and charting:

“*So*, *to start with it was a bit time consuming*. *Especially*, *for myself and the nurses as well”*

(ID 3 Male)

“*It took a lot longer because we were making so many mistakes*. *Because and plus you are looking at every single surfaces as well*, *so it used to get a bit you know*, *you could shout one code and your five codes ahead and your nurses like three codes behind*, *so it did get a bit confusing*.”

(ID 2 Femalea)

However, another participant expressed how patients becoming frustrated and external pressures were pivotal factors for dentists to base their decisions on:

“*I had to discontinue ICDAS because my patients were taking longer to deal with and because of the works strain*, *the rest of the waiting list was just getting impatient*. *So*, *I had to discontinue it*.”

(ID 3 Female)

Furthermore, many themes were found to be interrelated, but the complex issue that was identified was probably deficiency of training not only of the dentist during undergraduate studies but more especially amongst the dental nurses who had previously trained in a less time consuming method.

“*As some of them [nurses] didn’t come on the initial [research] trial training that as*, *as the dentist all of us went on and that wasn’t a fault of the trial*, *it was our trainer and the chap that owned the practice didn’t release everybody and sometimes they’d been put in and they were so used to charting one way and then we were expecting them to do something completely different and also it was trying to get them on board to help with the paperwork and the booklets that were being expected to be completed as well that*. *That I think they were used to working in a bit faster paced environment and maybe it slower things down a little bit*.”

(ID 4 Female)

“*I think*, *it’s more of a “faff” more than anything because you don’t get paid for prevention and ultimately*. *Yes*, *we do want to help patients*, *we do care about patients*, *but*, *at the end of the day it still is a job for us*.”

(ID 2 Femalea)

“*If it’s not going to benefit the patient or myself*, *in terms of making my job easier or making the treatment easier and it’s gonna take longer*. *Largely that’s not really gonna work*.”

(ID 4 Male)

#### Training

The participants recognised the significance of being used to a system through having been trained in it as an undergraduate and using it commonly in practice. In relation to ICDAS this was a significant issue. None of the participants, and more importantly none of their dental nurses, had been introduced to ICDAS during their clinical training and this was expressed as a significant barrier. A participant revealed:

“… *I think the main barriers probably it’s something that I wasn’t trained [as an undergraduate] with and I am more as familiar with other techniques and the same for the staff that haven’t worked with it too …”*

(ID 4 Female)

Positive feelings expressed by one participant explained that dentists were given sufficient training during the research. But, some of the dental nurses were not trained enough to ingrain the ICDAS system and were not convinced about using it:

“*I think because you have to have a trained nurse in it that’s very difficult*, *difficult to encourage some people certainly in the practice that I was in*. *Ahmm*, *so I think that would be probably barrier no*.*1*, *then after that it’s just getting used to it*. *Once you have had so much training in doing it [charting] one way*, *trying to do it in a different way is going to be quite difficult*.”

(ID 1 Female)

Participants were not only concerned about training, but commented that on a personal level characteristics of dental nurses such as years of experience and training using older/ other systems was a barrier to accepting the ICDAS system, though sometimes this was not related to the ICDAS codes but to other features of the dental charting such as unfamiliar tooth notation:

“*…*.*every nurse is different in amount of experience and training*. *I think some were sort of maybe not so well experienced with charting*. *And*, *I think they struggled with this little bit*. *So*, *I think some found it difficult because they weren’t familiar with e*.*g*. *more familiar with upper left 7 and not FDI notations in 4*, *7 and 4*, *8*.”

(ID 2 Male)

It was affirmed that the GDP’s may not consider a sudden transformation in practice, more focus on training was perceived important to alter their practices and accepting the system completely. This was evident when a participant stated:

“*But I think*, *for most of us who are just trained in dentinal caries*, *that might be a big thing*, *we might not want a change in that system so much*, *so maybe simplify the codes a little bit*. *Give us much more training*, *much more time onto it*. *So*, *we actually understand the codes a bit more*.”

(ID 2 Femalea).

The above mentioned quotes demonstrate that lack of simplicity is a noticed barrier amongst the general dental practitioners.

### Potential facilitators for increasing the usage of ICDAS by GDP’s

In addition to expressing their views on barriers of using the ICDAS system, participants suggested solutions or facilitators to help in uptake of the system by GDP’s in clinical settings and research. Most of the participants commented on making the ICDAS system computerised which would possibly lead to a wider majority willing to change their practice and increase their efficiency while using it.

“*Because*, *it’s all on the paper the charting was a bit difficult to deal with*. *And*, *I think so with the new technology*, *even the ICDAS should be computerised*.”

(ID 3 Female)

“*Yeah*, *I definitely think so because there are times that we had to start over with a new chart and things because there would be so many scribbles on it*, *which most of the time I had to redo in my spare time*. *Whereas*, *if it was computerized you could just double click and it would be sorted*.”

(ID 1 Female)

The participants also revealed that providing more training and skills to the dental health professionals (dentist and dental nurses) from a grass root level on the ICDAS system would help in overcoming the barrier of not understanding and the disadvantage of spending a lot of time. Consequently, they would become more skilful and faster in using it; their mind-set would change:

“*I think the main one is probably familiarization so I am used to doing it the way I was trained in university to do it*. *I do personally think that it takes longer*, *but I think if you did regularly have to use it you’d get quicker and you’d get more experienced in*, *that barrier maybe could be overcome…*.”

(ID 4 Female)

They also acknowledged that their use of the ICDAS system would have been a lot more efficient and pleasant in research, if they would have had additional instructions such as the use of a flow chart, pictures or having hands-on skills to make faster judgements of the caries codes. One of the participants stated:

“*…I personally found that really helpful [when shown the ICDAS decision tree]*, *like just working that logical way*, *so maybe having something like that available a flow chart that would help you*, *get you two numbers that you needed to so the result would be something I would have found useful…”*

(ID 4 Female)

It was pointed out by a participant that if there was former teaching on the ICDAS system at an undergraduate level, it would have had a positive impact on their learning experiences and would have advantaged patients by using it early on in their dental practice:

“*I would have liked to have had the training earlier because even in university because I found it quite helpful system for scoring caries and would have put it into my clinical practice earlier*.”

(ID 1 Female)

Even though most of the participants had not recently used the ICDAS system because of the aforementioned barriers, they did mention how useful it is in providing better prevention and dental care; they would use it in their clinical practices if the barriers were to be overcome.

“*I think it’s a really good way of charting because it is so thorough and it’s very systematic*, *so you know you are not missing any surface*. *Ahmm*, *which is a real benefit I think*, *it’s just so easy to skirt over the top of things*. *But*, *as I mentioned before there are some barriers to putting that in place*.”

(ID 1 Female)

“*I think it would be easy*, *it would be definitely be easy*, *if everybody uses it*.”

(ID 1 Male)

## Discussion

The aim of this study was to achieve a better understanding of the barriers and facilitators for GDP’s to use the ICDAS system in research and clinical practice. The literature review highlighted that there is a knowledge gap in the area of utilisation of the ICDAS system; therefore this study fills an evidence gap. There were four main themes identified as barriers complexity, cost implications, time disadvantage and training need and facilitators training, computerisation of the system, providing additional instructions that would lead to improving the utilisation of the ICDAS system in the future. The facilitators show that despite several barriers, the GDP’s want to provide prevention, better dental care and become more accustomed to the new.

This is the first study to identify barriers and facilitators explained by the GDP’s who have used the ICDAS system. The barriers and facilitators identified by the GDP’s can be mapped to the theoretical domain framework (TDF) which aids the understanding of the findings and perhaps suggests next steps in the implementation of ICDAS. The TDF framework identifies the possible influences that are involved with the behaviour of health professionals, explanation of influences and their related domain; provides techniques to bring about a behaviour change [29]. The TDF has 14 important domains i.e. ‘Knowledge’, ‘Skills’, ‘Social/Professional Role and Identity’, ‘Beliefs about Capabilities’, ‘Optimism’, ‘Beliefs about Consequences’, ‘Reinforcement’, ‘Intentions’, ‘Goals’, ‘Memory, Attention and Decision Processes’, ‘Environmental Context and Resources’, ‘Social Influences’, ‘Emotions’, and ‘Behavioural Regulation’ [30]. We distinguished 4 out of the 14 TDF (theoretical construct domains) (Table 3), of potential relevance based on the barriers and facilitators.

**Table 3:** Priority domains mapped from research findings for future investigations.

Domains identified	Barriers to be targeted	Facilitators for change
Knowledge	Lack of simplicity	Former teaching/use of the system (Undergraduate level)
Skills	Training	More practice-based training and skills
Environment context/ Resources	Financial implications	Incentives (for ICDAS and for caries prevention)Additional material for instructions(Training to improve speed)
Social influences	Time consuming (impact on other staff and patients)	Computerisation (Training/familiarisation to improve speed)

In this study the quantitative measure of ‘knowledge’ was not explored; instead it looked at the ‘perceived knowledge’ of the GDP’s about the ICDAS system. These participants confirmed previous research findings that undergraduate level instructions and opinions from academic experts have strong influences on health care professional’s behaviour on their future practices [31]. Moreover, opinions from perceived experts and colleagues could transform the intentions of GDP’s to utilize new technologies and practices, they were described as most important drivers for decision making by the participant’s; these factors may contribute to a underutilisation of the ICDAS system in clinical settings. Subsequently, we suggest that future studies take into account the differences in caries diagnosis practices in clinical practice between GDP’s with prior knowledge of the ICDAS system at an undergraduate level and those who started using the system at a later stage. Skills’ was expressed as an issue that affected the involvement of the GDP’s while utilising the ICDAS system in the previous trial and their clinical practice. The participants also agreed that it is important to provide extensive training for dental care professionals on the ICDAS system to be able to bring about a cultural shift in their caries diagnosis practices and the transformations from caries diagnosis norms is not possible solely by providing them with the knowledge. This may be due to the fact that change of behaviour of health care professionals cannot be brought by a one off step, it is a procedure that takes place over a period of time [32]. As a consequence, we suggest that future studies provide wide-ranging practical training on the ICDAS system to both dentists and dental nurses and provide adequate attention to the difference of skills between individuals.

This study confirmed ‘cost’ is a crucial factor for dentists while considering a new diagnostic test into dental practice which was consistent with the findings from Berwick and colleagues [33]. Insufficient financial incentives to using the ICDAS system was a repeated theme, indeed the additional time to complete an ICDAS chart was seen as a financial disincentive. From the participants it was expressed that financial benefit to dentists working in clinical practice settings was a foremost choice when it comes to choosing a caries diagnosis system, the ICDAS system had little or no cash value for them. Many participants felt uncomfortable with using and adopting the ICDAS system in clinical practice as the caries diagnosis procedure takes longer to use and the consequent cost of applying it is a strong consideration. In the study the incentive barrier went beyond payment and remuneration for GDP’s to adopting the ICDAS system, they also commented on the inadequate nature of reimbursement for the prevention of caries in general dental practice. As such they could not see a role for the discovery of initial caries lesions which did not require operative intervention, something which they would be paid for.

Material investments play a role while utilising ICDAS too. Participants indicated that they would have found the ICDAS system more beneficial, if they were given additional materials for instructions such as pictures or flow charts that would aid them in making quicker clinical judgements in their busy working day.

The fourth domain ‘social influences’ is a concept that states that individual thoughts are influenced by various factors such as peer pressure, opinions by colleagues, social norms and pressures from other groups [30]. This notion of ‘social influences’ is linked here with a major barrier ‘time’ found in the results. While ‘time’ as already discussed has impact on cost, there were other aspects related to time which emerged as being important; the impact on patients and other staff. From the interviews there was a common perception by the GDP’s regarding the time disadvantage while using the ICDAS system in the previous research; this shared perception influenced their future decision of not utilising the system in further clinical practice. The GDPs in this study were unaware of the available software for recording ICDAS electronically, as they had not used this within the trial in which they participated. One possible explanation about the reduced usage of the ICDAS system by the GDP’s is a concept called “Diffusion of innovations”, which suggest that an “innovation” is an idea or entity that is supposed to be new, while “diffusion” states the progressions through which an innovation spreads [34]. It is easy to comprehend that the innovators and the early adapters would potentially use the ICDAS system. Therefore, the rest of the proportion of the bell shaped curve as explained by the theory i.e. early majority and the late majority are either unaware or resistant to using the system.

The participants were also not aware of a much abbreviated version of ICDAS caries severity coding, Merged ICDAS, which has only 3 categories: initial, moderate and extensive caries severity [35]. This modification recognises not only the time constraints in clinical practice but also the issue identified with the complexity of the full six-stage ICDAS codes and that there is little clinical benefit in differentiating between the two earliest stages of enamel caries (ICDAS 1 and 2) which some practitioners reported as being difficult.

The study sample comprised of GDP’s who took part in a research trial in the past, this might have influenced the respondents’ recall of certain elements of the ICDAS system. There may also be an element the information they presented being affected by others within the previous study. For example, either a positive or negative experience within the previous trail may have influenced the challenges and the ease to which ICDAS’s use was reported by the participants. Measures were taken to lessen the recall bias by the respondents by providing them with sample charts from the previous research to prompt their thoughts. To reduce the homogeneity between GDP’s in this study there was some purposeful selection of interviewees ensuring a balanced representation by gender (four men and four women), a spread of ages (25–55 years) and years of experience (3–20 years in practice as GDPs) and with different areas of dental interests (4 GDP’s, 2 GDP’s waiting to work in speciality training of maxillofacial surgery, oral medicine and 2 GDP’s that had recently started working in community dental settings). Regarding the sample size, GDP’s from a previous research study were chosen using convenient but purposive sampling, it may have been useful and the findings might have varied if the study was to have interviewed a larger sample size of dentists from various work settings e.g. community general dental surgeons, hospital based dental surgeons, specialist trainees.

Moreover, the dependability of this study can be argued as the participants were GDP’s, they might not have wished to reveal their true weaknesses while using the ICDAS system for caries diagnosis process to the interviewer who was also a dentist. This bias might have influenced the findings in this study. However, the interviews were conducted in their personal dental practices, so that external influences could be avoided while they answered. If the participants would have been interviewed for a longer period of time, a more in-depth conversation with detailed findings might have been found. However, an attempt was made to reduce the influence of this drawback by conducting thematic analysis by Braun and Clarke’s method. An attempt was made to reduce this interviewer bias from this study by asking the same open ended questions from all the participants and increasing comparability between their answers; reaching a point of saturation. The investigator was given prior training on how to conduct neutral interviews without imposing their own pre-conception.

Questions can be raised about the cause and effect interferences in this study due to the sample size not being large enough for the findings to be generalised. However, unlike quantitative research where a large sample size is needed for generalisability, in an qualitative research it is fundamental to explore meaning in depth rather than quantifying them [36]. Most of the research around qualitative interview based studies proposes transferability and dependency, rather than generalisability.

## Conclusion

This qualitative exploratory research study took place with UK-based dentists who had been working in general dental practice at the time of being involved in research utilising ICDAS; it was designed to explore the views and opinions of selected GDP’s regarding barriers and facilitators while using the ICDAS system in previous research. The findings reveal that GDP’s understand the important role the ICDAS system plays in caries prevention but there are various obstacles faced by the GDP’s while implementing the system into practice; also, there are possible drivers that are likely to overcome those barriers and increase the acceptance of the system. The research provided insights of the GDP’s, many facilitating factors such as making the ICDAS system computerised, giving additional training on the system to the GDP’s and dental nurses, ensuring undergraduate level teaching on the system (former teaching), additional incentives for using the system and for rewarding caries-preventive practices, could help in increasing the utilisation of the ICDAS system in clinical practice. Based on Rogers Diffusion of Innovation 2008, the adoption of new technologies is not a simple straightforward process it is a multi-level process that is influenced by contextual factors, national policies, organisational issues and adopter’s individual characteristics [34]. Thus, there is a need for the wider agencies such as the oral health service planners, policy makers and stakeholders to design a plan to implement the ICDAS system in clinical practice in a more acceptable way to the GDP’s. For example, if a corporate body such as British United Provident Association (BUPA) said it was their policy to do an ICDAS chart for every new patient, then their dental practices would automatically adopt the practice because that would be a requirement of the body commissioning their service in this way their use of ICDAS would be supported and facilitated by the policy.

## Supporting information

S1 File Interview transcripts. (ZIP)

S1 Fig Thematic map. (TIFF)

S1 Table COREQ Checklist. (DOC)

## References

[1] Featherstone JDB. Prevention and reversal of dental caries: role of low level fluoride. Community Dentistry and Oral Epidemiology. Wiley; 1999;27: 31–40. 10.1111/j.1600-0528.1999.tb01989.x 10086924

[2] Kassebaum NJ, Bernabé E, Dahiya M, Bhandari B, Murray CJL, Marcenes W. Global Burden of Untreated Caries. Journal of Dental Research. SAGE Publications; 2015;94: 650–658. 10.1177/0022034515573272 25740856

[3] Ismail AI, Sohn W, Tellez M, Amaya A, Sen A, Hasson H, et al The International Caries Detection and Assessment System (ICDAS): an integrated system for measuring dental caries. Community Dentistry and Oral Epidemiology. Wiley; 2007;35: 170–178. 10.1111/j.1600-0528.2007.00347.x 17518963

[4] Ismail AI. Visual and Visuo-tactile Detection of Dental Caries. Journal of Dental Research. SAGE Publications; 2004;83: 56–66. 10.1177/154405910408301s12 15286124

[5] Bader JD, Shugars DA, Bonito AJ. A systematic review of selected caries prevention and management methods. Community Dentistry and Oral Epidemiology. Wiley; 2001;29: 399–411. 10.1034/j.1600-0528.2001.290601.x 11784283

[6] Dikmen B. ICDAS II CRITERIA (INTERNATIONAL CARIES DETECTION AND ASSESSMENT SYSTEM). Journal of Istanbul University Faculty of Dentistry. Istanbul University; 2015;49: 63 10.17096/jiufd.38691 28955548 PMC5573507

[7] Topping GVA, Pitts NB. Clinical Visual Caries Detection. Monographs in Oral Science. KARGER; 2009 pp. 15–41. 10.1159/000224210 19494673

[8] Shivakumar K, Prasad S, Chandu G. International Caries Detection and Assessment System: A new paradigm in detection of dental caries. Journal of Conservative Dentistry. Medknow; 2009;12: 10 10.4103/0972-0707.53335 20379434 PMC2848805

[9] Anderson P, Beeley J, Monteiro PM, de Soet H, Andrian S, Amaechi B, et al A European Core Curriculum in Cariology: the knowledge base. European Journal of Dental Education. Wiley; 2011;15: 18–22. 10.1111/j.1600-0579.2011.00709.x 22023542

[10] Nunn J, Morris J, Pine C, Pitts N, Bradnock G, Steele J. The condition of teeth in the UK in 1998 and implications for the future. British Dental Journal. Springer Nature; 2000;189: 639–644. 10.1038/sj.bdj.4800853 11191176

[11] SHEIHAM A. Changing Trends in Dental Caries. International Journal of Epidemiology. Oxford University Press (OUP); 1984;13: 142–147. 10.1093/ije/13.2.142 6376384

[12] ARTA G, CAGETTI MG, COCCO F, SALE S, LINGSTRÖM P, CAMPUS G. Caries-risk profiles in Italian adults using computer caries assessment system and ICDAS. Brazilian Oral Research. FapUNIFESP (SciELO); 2015;29 10.1590/1807-3107bor-2015.vol29.0126 26892361

[13] de Amorim RG, Figueiredo MJ, Leal SC, Mulder J, Frencken JE. Caries experience in a child population in a deprived area of Brazil, using ICDAS II. Clinical Oral Investigations. Springer Nature; 2011;16: 513–520. 10.1007/s00784-011-0528-9 21384127 PMC3307999

[14] Braga MM, Oliveira LB, Bonini GAVC, Bönecker M, Mendes FM. Feasibility of the International Caries Detection and Assessment System (ICDAS-II) in Epidemiological Surveys and Comparability with Standard World Health Organization Criteria. Caries Research. S. Karger AG; 2009;43: 245–249. 10.1159/000217855 19439944

[15] Honkala E, Runnel R, Honkala S, Olak J, Vahlberg T, Saag M, et al Measuring Dental Caries in the Mixed Dentition by ICDAS. International Journal of Dentistry. Hindawi Limited; 2011;2011: 1–6. 10.1155/2011/150424 22114594 PMC3206401

[16] Braga MM, Mendes FM, Ekstrand KR. Detection Activity Assessment and Diagnosis of Dental Caries Lesions. Dental Clinics of North America. Elsevier BV; 2010;54: 479–493. 10.1016/j.cden.2010.03.006 20630191

[17] Kühnisch J, Berger S, Goddon I, Senkel H, Pitts N, Heinrich-Weltzien R. Occlusal caries detection in permanent molars according to WHO basic methods, ICDAS II and laser fluorescence measurements. Community Dentistry and Oral Epidemiology. Wiley; 2008;36: 475–484. 10.1111/j.1600-0528.2008.00436.x 18422704

[18] Mehta A. Comprehensive review of caries assessment systems developed over the last decade [Internet]. 2012 [cited 6 August 2019]. http://vdisk.univille.edu.br/community/depto_odontologia/get/ODONTOLOGIA/RSBO/RSBO_v9_n3_julho-setembro2012/v9n3a14.pdf

[19] Gomez J, Ellwood RP, Martignon S, Pretty IA, Gomez Bulla J. Dentists’ perspectives on caries-related treatment decisions. Community Dental Health. 2014;31(2):91–98. 10.1922/CDH_3341Gomez08 25055606

[20] Bader JD, Shugars DA. Variation in Dentists’ Clinical Decisions. Journal of Public Health Dentistry. Wiley; 1995;55: 181–188. 10.1111/j.1752-7325.1995.tb02364.x 7562733

[21] Pitts NB, Richards D. Personalized Treatment Planning. Monographs in Oral Science. KARGER; 2009 pp. 128–143. 10.1159/000224217 19494680

[22] Deery C. Caries detection and diagnosis, sealants and management of the possibly carious fissure. British Dental Journal. Springer Science and Business Media LLC; 2013;214: 551–557. 10.1038/sj.bdj.2013.525 23744208

[23] Luz PB, Stringhini CH, Otto BR, Port ALF, Zaleski V, Oliveira RS, et al Performance of undergraduate dental students on ICDAS clinical caries detection after different learning strategies. European Journal of Dental Education. Wiley; 2014;19: 235–241. 10.1111/eje.12131 25495379

[24] Almerich-Silla J, Boronat-Ferrer T, Montiel-Company J, Iranzo-Cortes J. Caries Prevalence in Children from Valencia (Spain) using ICDAS II criteria, 2010. Medicina Oral Patología Oral y Cirugia Bucal. Medicina Oral, S.L.; 2014; e574–e580. 10.4317/medoral.19890 25350591 PMC4259373

[25] El-Damanhoury H, Fakhruddin K, Awad M. Effectiveness of teaching International Caries Detection and Assessment System II and its e-learning program to freshman dental students on occlusal caries detection. European Journal of Dentistry. Georg Thieme Verlag KG; 2014;8: 493 10.4103/1305-7456.143631 25512730 PMC4253105

[26] Norlyk A, Harder I. What Makes a Phenomenological Study Phenomenological? An Analysis of Peer-Reviewed Empirical Nursing Studies. Qualitative Health Research. SAGE Publications; 2010;20: 420–431. 10.1177/1049732309357435 20068190

[27] Braun V, Clarke V. Using thematic analysis in psychology. Qualitative Research in Psychology. Informa UK Limited; 2006;3: 77–101. 10.1191/1478088706qp063oa

[28] Miles M, Huberman A. Qualitative data analysis an expanded sourcebook. Thousand Oaks: SAGE; 1994.

[29] Francis JJ, O’Connor D, Curran J. Theories of behaviour change synthesised into a set of theoretical groupings: introducing a thematic series on the theoretical domains framework. Implementation Science. Springer Nature; 2012;7 10.1186/1748-5908-7-35 22531601 PMC3444902

[30] Cane J, O’Connor D, Michie S. Validation of the theoretical domains framework for use in behaviour change and implementation research. Implementation Science. Springer Nature; 2012;7 10.1186/1748-5908-7-37 22530986 PMC3483008

[31] Locock L, Dopson S, Chambers D, Gabbay J. Understanding the role of opinion leaders in improving clinical effectiveness. Social Science & Medicine. Elsevier BV; 2001;53: 745–757. 10.1016/s0277-9536(00)00387-7 11511050

[32] Fitzgerald L, Ferlie E, Wood M, Hawkins C. Interlocking Interactions, the Diffusion of Innovations in Health Care. Human Relations. SAGE Publications; 2002;55: 1429–1449. 10.1177/001872602128782213

[33] Berwick DM, Nolan TW, Whittington J. The Triple Aim: Care, Health, And Cost. Health Affairs. Health Affairs (Project Hope); 2008;27: 759–769. 10.1377/hlthaff.27.3.759 18474969

[34] Rogers EM. Evolution: Diffusion of Innovations. International Encyclopedia of the Social & Behavioral Sciences. Elsevier; 2015 pp. 378–381.

[35] Pitts N, Ekstrand K. International Caries Detection and Assessment System (ICDAS) and its International Caries Classification and Management System (ICCMS)—methods for staging of the caries process and enabling dentists to manage caries. Community Dentistry and Oral Epidemiology. Wiley; 2013;41: e41–e52. 10.1111/cdoe.12025 24916677

[36] Leung L. Validity, reliability, and generalizability in qualitative research. Journal of Family Medicine and Primary Care. Medknow; 2015;4: 324 10.4103/2249-4863.161306 26288766 PMC4535087

