# Supplementary material for: The views and experiences of general dental practitioners (GDP’s) in West Yorkshire who used the International Caries Detection and Assessment System (ICDAS) in research
Source: PLoS One. 2019 Oct 4;14(10):e0223376. doi: 10.1371/journal.pone.0223376 (PMC6777823; doi:10.1371/journal.pone.0223376)
Supplement: S1 File — (ZIP) [file pone.0223376.s001.zip › Transcripts/Transcript 1.docx]

Interviewer: Right then, Sir can you please tell Interviewer about your use of ICDAS in the research and how many tiInterviewers approximately you have done it in research?

ID 1 Male: Well I was, a part of the my practice was a part of the, what do you call it, it was a part of the, what to do, of a research program, which was West Yorkshire wide and we were supposed to look at the patients and see the caries and record it according to the ICDAS system. And then review them every six months or a year. According to the patients, ahhhh their risk level and then see whether it’s getting better or not? You know. So, how many tiInterviewers I used it? We used it for I think more than about 40-50 patients, more than 50 patients.

Interviewer: And, if you could change your ICDAS experience, what changes would you make?

ID 1 Male: ICDAS experience, Interviewerans basically ICDAS, what I understood was which was 3 years ago when I last used it. Looking at the tooth and looking at without radiographs. And classifying whether how much, what is the extent of dental caries in that tooth and then making a treatInterviewernt plan accordingly. And, what I would change? Nothing, I think it is a very good system.

Interviewer: Okay, since the research has ended have you used ICDAS system in clinical practice?

ID 1 Male: No.

Interviewer: Alright, has the training influenced your clinical diagnosis and treatInterviewernt of patients.

ID 1 Male: Yes, that is interesting because the ICDAS training which was given by the trainer [refers to a professor at the university]. I think was very good and that is actually, we don't use it as ICDAS but we can now look at the tooth and find out according to the opacity and according to the black areas, whether how much deep the caries would be and then we usually ratify that with taking radiographs.

Interviewer: What system do you normally use in dental practice to detect caries?

ID 1 Male: We use Tactile, Visual and Radiographic.

Interviewer: And, how often do you use it in your dental practice, the system that you normally use?

ID 1 Male: There is no limit, every patient.

Interviewer: Every patient?

ID 1 Male: Every patient, 40 tiInterviewers a day.

Interviewer: Was there a cultural shift from your normal caries diagnosis practice and using ICDAS?

ID 1 Male: It was, definitely, definitely, because it did not ask us to use any probes or anything. So, and the patients was in the beginning were very surprised that we were not using any instruInterviewernts while looking at the teeth. Just drawing, drying the tooth and having a look at it and making a decision.

Interviewer: So, how did the patients react and feel or did they, did they not notice a change in caries assessInterviewernt process?

ID 1 Male: They did notice, but they were fine with it. They did not, they were not anti-it or they did not want to go back to the old system or wanting to know why you have changed it? Nothing, but they felt the difference.

Interviewer: And, how did the dental nurses feel about the change in caries assessInterviewernt process?

ID 1 Male: They were fine, but they were on the receiving end because we were just speaking and they had to write it down and they had to note it down. So, it was an extra work for them because the, the computer programs we were using for recording for our patients record did not have the ICDAS system in it. So, it could not be recorded on the computer straight. And extra work for them.

Interviewer: If, why wouldn’t you use ICDAS in your dental practice?

ID 1 Male: Why? That is an interesting question, why? Because, it is not mainstream. So, everybody is not using it. It is not comparable. So, if in one practice for example here we are 6 dentists and if 1 person is using ICDAS and the patients usually stay with one dentist but if they go to the other dentist and if there is a bit of discrepancy, the patients may not be very happy. So, ICDAS should be used across the board and then everybody will be more happy. So, it is like using 2-3 different systems.

Interviewer: Do you think it would be easy to use in practice?

ID 1 Male: I think it would be easy, it would be definitely be easy, if everybody uses it.

Interviewer: And, could you please recall and tell Interviewer about the difficult codes in ICDAS.

ID 1 Male: The easy answer is, No there is nothing difficult in it.

Interviewer: There is nothing difficult in it?

ID 1 Male: No.

Interviewer: Alright, So and what about, can you please tell Interviewer about the charting quality. Is there anything which might have affected the quality of the charting?

ID 1 Male: No, I think everything was quite simple, quite straightforward. Especially the training package is very good. So, you go through it a couple of tiInterviewers and then you do the, you look at the patients mouth surface. It is very simple.

Interviewer: Alright, then that’s it. Thank you very much.

ID 1 Male: That’s good
