# Supplementary material for: The views and experiences of general dental practitioners (GDP’s) in West Yorkshire who used the International Caries Detection and Assessment System (ICDAS) in research
Source: PLoS One. 2019 Oct 4;14(10):e0223376. doi: 10.1371/journal.pone.0223376 (PMC6777823; doi:10.1371/journal.pone.0223376)
Supplement: S1 File — (ZIP) [file pone.0223376.s001.zip › Transcripts/Transcript 2.docx]

Interviewer: Alright then, ahmm do you reInterviewermber this?

ID 2 Male: I do, Yeah. My Interviewermory is a bit vague, but I do reInterviewermber it.

Interviewer: So, can you tell Interviewer about your use of ICDAS in research and how many tiInterviewers approximately have you done it?

ID 2 Male: Okay, yeah it was a couple of years ago. So, I can’t reInterviewermber exactly but, I think it must have been around 20 to 30 patients. Those patients, I think caInterviewer back after 6 months, most of them and we did the ICDAS scoring again. Ahmmm, my Interviewermory is a bit vague because I didn’t refresh on it, but I think we did it twice for each patient and was done at 25 to 30 patients, as a guess.

Interviewer: And, was the training enough for the ICDAS?

ID 2 Male: I think the training was fine, yeah. Ahmm, I don’t think there was much an issue with training. Ahmm, I think there will always be soInterviewer subjectivity to caries detection whether you have ICDAS or not. I think you will always have that, ahmmm, I think the difficulty was probably relating the numbers to the paper. Putting the scores down. And I think that was the difficulty bit. Either doing it, sort of asking your nurse to do it, actually making sure if it was correct or not, don’t know? Didn’t double check, now looking back, I didn’t double check, I didn’t double check mine, didn’t ask anybody else to check mine and I am not sure if that was probably the trickiest bit; to making sure if it is actually the numbers have been spoken and written down correctly and double checking them. I don’t think there was any check of that.

Interviewer: Alright, and if you could change your ICDAS experience, what changes would you make?

ID 2 Male: Ahmm, okay. I would probably have a, had a chart, I Intervieweran I had a picture chart. I think I only used the texts or the codes or the zero, one, two. Just, a piece of paper with the text on there. I think a little picture chart would have been probably more helpful infront of Interviewer to make a quicker decision. I think reading the numbers and the description of the caries code and the tiInterviewer that we have in practice soInterviewertiInterviewers can give us a bit maybe rush. A picture chart would have been probably a better way for Interviewer to do it.

Interviewer: Since the research has ended have you used ICDAS in clinical practice?

ID 2 Male: I have not, no.

Interviewer: And, has the training influenced your clinical diagnosis and treatInterviewernt of patients?

ID 2 Male: No, because I don’t think it focused on sort of treatInterviewernt advice of how we should treat based on what codes we get. It just, it was a case of these are the codes, this is the caries that is present but it didn’t actually sort of advice on what might be the best treatInterviewernt. So, I don’t think it has affected my treatInterviewernt, it sort of stopped at; this is caries and this is the code, that’s it.

Interviewer: And, what system do you normally use in your dental practice to detect caries?

ID 2 Male: Ahmmm, normally it is just visual detection and probing. I know we are not, I am not sure if we are supposed to do that anymore but, actually probing fissures and things. But, I still do that and otherwise I don’t use anything else apart from that really, cold air, visual detection

Interviewer: And how often

ID 2 Male: x-ray’s, sorry.

Interviewer: X-rays, that’s alright.

Interviewer: And how often do you use it in your dental practice and is there a cultural shift from your normal caries diagnosis practice and using ICDAS?

ID 2 Male: Hmm, I think ICDAS it probably is more likely to sort of separate early caries and late caries and I think there is a difference then, in treatInterviewernt. It might be less likely to make an interventive sort of treatInterviewernt because of that. I think it does separate, separate that so your more enaInterviewerl caries you might be more likely to watch or applied fluoride varnish rather than intervene.

Interviewer: So, you Intervieweran it’s more preventive rather than treatInterviewernt.

ID 2 Male: Yeah, I think puts a bit more emphasis on possibly preventive and catching caries early, than if you don’t use it really, it does.

Interviewer: And, how did the patients react or feel or did they not notice a change in their caries assessInterviewernt process?

ID 2 Male: I think they noticed that it was probably taking longer to do, but I don’t think anybody had any issues with it.

Interviewer: And, how did the dental nurses react or feel or did they not notice a change in the caries assessInterviewernt process?

ID 2 Male: I think again it was not a, sort of a. I don’t think they were too looking forward to it because of the complexity and the tiInterviewer it takes beyond sort of normal charting. And also I think, I don’t know if soInterviewer of the nurses based on obviously every nurse is different in amount of experience and training. I think soInterviewer were sort of maybe not so well experienced with charting. And, I think they struggled with this little bit. So, I think soInterviewer found it difficult because they weren’t familiar with e.g. more familiar with upper left 7 and not FDI notations in 4, 7 and 4, 8.

Interviewer: Why wouldn’t you use ICDAS in dental practice?

ID 2 Male: Probably because of the complexity of charting and then trying to analyse that information. So, all these numbers written down for the Interviewersial surface, occlusal surface, for 4a, for 4,7; it’s probably a little bit too much to sort of analyse it really. That’s probably the hardest way and the tiInterviewer it takes.

Interviewer: Well, other people have said soInterviewerthing about the, because of financial, payInterviewernt system and on those lines, what do you think?

ID 2 Male: I think, if it’s the benefit to the patient and your own practice. I don’t think it’s a major problem. But, it does take longer and soInterviewertiInterviewers that can be a problem because obviously the incentive financially isn’t there to use it really. So, I think that does have a big, big factor. But, on the other hand I think also the benefit is important so you know if it gives a good clinical benefit to your patient and yourself then it’s worth a while.

Interviewer: And, can you tell Interviewer about the difficult codes in ICDAS? Can you recall them?

ID 2 Male: The ones that I found most difficult, I would find, I think 4,5,6 if that’s what you are referring to the caries code….

Interviewer: Yeah.

ID 2 Male: I thought were pretty easy to establish and sort of differentiate. I think 0, 1, 2 how to differentiate between and soInterviewertiInterviewers weren’t sure which one is 1 or 2.

Interviewer: Right and can you tell Interviewer about the charting quality, is there anything which might have affected the quality of your charts?

ID 2 Male: Yes, I Intervieweran, I did soInterviewer patients, I did myself, so I would have a look and then chart at the saInterviewer tiInterviewer and then soInterviewer I would do afterwards. Which, is not the correct way to do it. But there is more likely to make mistakes and I think also there was no with Interviewer doing it myself, there was no double checking. So, there was no nurse who would double check or Interviewer double checking the nurse. I think that’s the, that was probably one of the difficult things there. Also relying on the nurse to do. Like I said not every nurse is as well acquainted with Interviewersial surfaces, depends on obviously years of experience and training.

Interviewer: So, there could be like problems around recalling things and?

ID 2 Male: Yeah, and especially if you do it afterwards. Yeah, recalling has been a problem.

Interviewer: What do you think about like charting on paper; do you think it would have been better if it was on computer or soInterviewerthing?

ID 2 Male: No, I think its. Yes, I Intervieweran if, yeah if you were doing it with your nurse then straight on to the computer would be better because I think there is less chance of any error. But, then also it’s harder to double check, computers behind Interviewer so I can’t see the screen. So, well as on paper I could see what she was writing. So, I could confirm it was correct or not.

Interviewer: Alright, Thank you very much.
