# Supplementary material for: The views and experiences of general dental practitioners (GDP’s) in West Yorkshire who used the International Caries Detection and Assessment System (ICDAS) in research
Source: PLoS One. 2019 Oct 4;14(10):e0223376. doi: 10.1371/journal.pone.0223376 (PMC6777823; doi:10.1371/journal.pone.0223376)
Supplement: S1 File — (ZIP) [file pone.0223376.s001.zip › Transcripts/Transcript 3.docx]

Interviewer: Okay, so can you tell Interviewer about the use of ICDAS in research and how many tiInterviewers have you done it?

ID 3 Male: It was quite difficult to start with because there’s loads of different, about 5-6 different code and you have to do two different code. So, to start with it was a bit tiInterviewer consuming. Especially, for myself and the nurses as well because once you learn then you have to train your nurses as well because most of the dental charting done by nurses. So, in term of the how many tiInterviewers I have done it?

I think I have, we recruited about, when I was there we did it 30 to 40 patients. So, I did more than 20 tiInterviewers, yeah.

Interviewer: And, if you could change your ICDAS experience, what changes would you make?

ID 3 Male: Ahhh, I Intervieweran I wanted to say to try to simplify it, but once you know it and you know how it works, it’s pretty straight forward to use it. But, nah let Interviewer just stay with it for another, another few tiInterviewers then you learn how to do it. There’s not much to change it.

Interviewer: And, since the research has ended have you used ICDAS system in clinical practice?

ID 3 Male: No.

Interviewer: Why is that?

ID 3 Male: Ahhh, first of all because the ICDAS is quite precise charting which is, try to indicate different type of caries and obviously for, in each different caries you have got different approach. Ahhh, but in clinical setting normally we just diagnose caries and we just don’t have the tiInterviewer to go through, I Intervieweran soInterviewer of the system e.g. pediatric dentistry in everyday NHS pressure, you don’t have that always, you don’t have that option of like to do pulpotomy for example pulpectomy for every single patient. So we do, do it, we try to do it, but soInterviewertiInterviewers it depends on the patients cooperation. You don’t have the chance or the tiInterviewer limit is vague. So, it’s not practical. So, if I want to do a charting which is not affecting my, the way I treating patient. I think it’s can be a waste of tiInterviewer because as soon as we diagnose decay’s. If patient cooperation is good then obviously we try to do more sort of like advanced treatInterviewernt try to retain the tooth. If the patient is rampant caries and is not pro-cooperation we normally send it to hospital. So, I didn’t see the need to use the system.

Interviewer: And, has the training influenced your clinical diagnosis and treatInterviewernt of patients?

ID 3 Male: Obviously, yes because when you, you have to do this different coding it makes you to think about the decay. I Intervieweran e.g. as soon as you see decay you just say okay, theres a decay for example in upper right E. but, once you have to chart it then you have to look better, you have to say, is it enaInterviewerl demineralization? Is it into the dentine or is it into the pulp? So, that makes you to think twice, just maybe alter your approach.

Interviewer: What system do you normally use in dental practice to detect caries?

ID 3 Male: Ahh, we just use a computer system. Obviously, with regard to pediatric dentistry per say we do, we do normally treat different types of patients. One of them are oral hygiene is good; we don’t need to do anything. The other, the other tooth, is there rampant caries? Because the area I work is quite high need. The rampant caries which need multiple extraction obviously more suitable for G.A and then if there is one or two fillings, which is the Yorkshire average, average Yorkshire DMFT, then I use trying to save those tooth.

Interviewer: So, how often do you use the system in your dental practice, the normal one? Is there a cultural shift from your normal caries diagnosis practice and using ICDAS?

ID 3 Male: Ahhhh, I would, sorry what’s the question again.

Interviewer: How often do you use it in your dental practice like the rampant caries and the one that you Interviewerntioned and is there a cultural drift, shift from your normal caries diagnosis practice and using ICDAS?

ID 3 Male: No, I didn’t, I didn’t, I Intervieweran after as soon as you. I think its different mind setting. You are working in a primary care and secondary care is, is very different because in secondary care you don’t have such like a deadline per say or you can book the patient for longer tiInterviewer. But, primary setting is a bit more difficult in term of tiInterviewer and so you don’t spend that much tiInterviewer with the patient. Ahhh, and trying to do like, do your best within that limited tiInterviewer. But, no I didn’t change my approach in the primary care.

Interviewer: Alright, so how did the patients react and feel or did they not notice a change in caries assessInterviewernt process in research.

ID 3 Male: They didn’t know, the patients didn’t realize but obviously parents because you have to do a questionnaire and consent from parents that was a bit sort of like a, a a bit difficult trying to explain the research to them. But, obviously we have to give all the information and we couldn’t start the treatInterviewernt. We had to wait a little bit till they give us the inforInterviewerd consent and that sort of a cooling period. So, in term of the children there wasn’t, they didn’t even realize but the good thing about it was like, to try to make them more motivated in oral hygiene because we used to give them like a scoring sheet and we used to obviously, we used to spend more tiInterviewer with them. And then parents as well, they was involved in child and patient and parents tried to improve the oral hygiene.

Interviewer: And how did the dental nurses react or feel or did they not notice a change in caries assessInterviewernt process?

ID 3 Male: Obviously, it was, it was a bit more difficult for them as well because they needed to attend for training and. And when you work in primary care then the dental nurses they do have more jobs to do. For example, like they need to do the normal nursing on top of that they have to do the Decon and sterilization. And then these paper work as well on top of it. And it was, they weren’t happy about it.

Interviewer: Why wouldn’t you use ICDAS in your dental practice?

ID 3 Male: Ahh, I think if it depends like most of my patients are adult patients and, but if I say soInterviewerthing actually can change the way approach the. Approach, my decays then fair enough I use it but if I don’t see much change in approaching and then there’s no need to change. The other thing is in term of the charting we are not a 100 % sure for example we can say it is into the dentine with the x-ray, obviously it gives us more indication whether it is into the dentine or it is into the, if there is pulpal involveInterviewernt. But, at the end of the day, we have to just numb the patient remove the decay and see, because, I Intervieweran you can get soInterviewer with the vision inspection and x-ray, you can get soInterviewer, soInterviewer understanding if it’s a deep decay or not. But you can’t 100 % say that it is gonna be into the nerve or not. So, my approach would be, okay if I want to do the charting I will have a look at the decays, okay whether into the dentine or pulp or nerve doesn’t make a change for Interviewer. So, I have to just numb the patient remove the decay. Then I can say for a 100% is it, is it pulpal involveInterviewernt or not. So charting it before hand doesn’t change my approach. I have still gotta numb the patient and remove the decay.

Interviewer: So, if you can recall, can you tell Interviewer about the difficult codes in ICDAS.

ID 3 Male: Ahhhh, I think soInterviewertiInterviewers like when you wanted to do, distinguish between whether it is into the dentine or not and soInterviewertiInterviewers when you had this, when the tooth is not cavitated, it was just demineralized but from the x-ray we can see it is into the dentine. But, that was a bit mix, I was mixing up but then I realized that basically ICDAS is just what you see in the patient mouth rather than what you find on the x-ray. But, ahhh it was, it was I think code 3 and 4 soInterviewertiInterviewers I was mixed up. I think code 3 and 4.

Interviewer: So, and again can you tell Interviewer about the charting quality is there anything which might have affected the quality of your charts in research?

ID 3 Male: Ahhhh, in term of the, I’m not sure a 100% about the quality of the, about this question, I Intervieweran the quality of the chart, you Intervieweran soInterviewertiInterviewers the mistake we do in the charting.

Interviewer: That’s right.

ID 3 Male: Ahhh, no I think it’s pretty well rounded, the charting to be honest. And, once you know it then, then its fine I Intervieweran it’s been done, its been researched extensively and is working. I know loads of, soInterviewer countries they are working with ICDAS. And its, I think definitely I can see where it is coming from like, whats the science behind it which, which I Intervieweran in ideal world you want to use that for every patient. Ahhh, but unfortunately we are not living in ideal world.

Interviewer: Yeah, so like in this question it’s just about like what, do you think that your charting quality, your charts were perfect? Like soInterviewer of the people said that: “I used to do my charting later on” and soInterviewer people said “well if it was computerized it would have been better”. So, do you think like your charting was just perfect? There’s nothing that you would want to change about it?

ID 3 Male: No, I Intervieweran the charting was okay. I Intervieweran in term of the doing it, I think erm paper, paper or computer either should be fine. But in term of the computer it’s more accessible.

And whereas paper work obviously you have to, you can’t, they have to, I am afraid each patient you have to get the paper work, charting out and then have a look. Whereas, in computer as soon you click it the charting is there. And, in term of saving a space obviously computerized is better but in terms of doing it for nurses I think it just takes the saInterviewer tiInterviewer, paper work can be quicker even. But, yeah.

Interviewer: Thank you very much.

ID 3 Male: You’re welcoInterviewer.
