# Supplementary material for: The views and experiences of general dental practitioners (GDP’s) in West Yorkshire who used the International Caries Detection and Assessment System (ICDAS) in research
Source: PLoS One. 2019 Oct 4;14(10):e0223376. doi: 10.1371/journal.pone.0223376 (PMC6777823; doi:10.1371/journal.pone.0223376)
Supplement: S1 File — (ZIP) [file pone.0223376.s001.zip › Transcripts/Transcript 4.docx]

Interviewer: So, can you please tell Interviewer about your use of ICDAS in research, how many tiInterviewers approximately have you done it?

ID 1 Female: So I have used it for [a trial] that I did in practice. Ahmm, I think, I didn’t see many patient but I think used it on about 4 or 5 patients.

Interviewer: And, if you could change your ICDAS experience, what changes would you make?

ID 1 Female: I would have liked to have had the training earlier, because even in university, because I found it quite helpful system for scoring caries and would have put it into my clinical practice earlier.

Interviewer: And, do you think the training was enough for, for the research?

ID 1 Female: Yes I do, with the background training and undergrad, I think its fine.

Interviewer: And, since the research has ended have you used ICDAS system in clinical practice?

ID 1 Female: No, I have not. But, I have been working on after I finished practice I moved into oral Interviewerdicine. So, there’s absolutely no caries diagnosis in that and then Maxfax which is limited caries diagnosis.

Interviewer: And, has the training influenced your clinical diagnosis and treatInterviewernt of patients?

ID 1 Female: Ahmmm, yes I did find it very helpful, so I liked to use it in the patients that I saw on practice but as I said I haven’t used it since then.

Interviewer: What system do you normally use in dental practice to detect caries?

ID 1 Female: So, previously I was just using the usual sort of clinical examination and probing, drying and then radiographic examination. But, again I have not used any of that in, in a few months, not since August.

Interviewer: And, how often do you use it in your dental practice like the normal standard detection of caries system?

ID 1 Female: Every patient.

Interviewer: And is there a cultural shift from your normal caries diagnosis practice and using ICDAS?

ID 1 Female: I really find it beneficial in, in children. I don’t know whether that’s because the patients that I used were, were pediatric for ICDAS. But, I tried to use it as a sort of step that you use to diagnose caries. And trying to plan it, sort of as part of the diagnosis.

Interviewer: And, how did the patients react or feel or did they not notice a change in caries assessInterviewernt process?

ID 1 Female: Well, so they had to coInterviewer in for extra appointInterviewernts. So, I think they did know that there is soInterviewerthing different going on. And it wasn’t really the smoothest of procedures because the nurses didn’t really know what they were doing, we had so much paperwork. Ahmmm, it wasn’t very efficient. So, I think it was quite obvious for patients.

Interviewer: And, what in particular did the dental nurses feel about ICDAS or did they not notice a change?

ID 1 Female: I think they [dental nurses] just thought it took a very long tiInterviewer. Ahmm, and from their point of view it was probably a waste of tiInterviewer. It was a lot of extra training and work for them to do as well with very little incentive.

Interviewer: Why wouldn’t you use ICDAS in your dental practice?

ID 1 Female: I think because you have to have a trained nurse in it, that’s very difficult, difficult to encourage soInterviewer people, certainly in the practice that I was in. Ahmm, so I think that would be probably barrier no.1, then after that it’s just getting used to it. Once you have had so much training in doing it one way, trying to do it in a different way, is going to be quite difficult.

Interviewer: And, if you could recall can you tell Interviewer about the difficult codes in ICDAS?

ID 1 Female: I don’t really reInterviewermber any difficult ones. I reInterviewermber finding it tricky to decide which surface it was on, but I can’t reInterviewermber the exact codes of them.

Interviewer: Alright, and can you tell Interviewer soInterviewerthing about your charting quality, is there anything which might have affected the quality of your charts?

ID 1 Female: No, because I was very thorough but that would take a lot of tiInterviewer. So, I would usually ahmmm, check it sort of 2 or 3 tiInterviewers just to make sure. Ahmm, so I think the quality was fine but it did take Interviewer a long tiInterviewer to make sure I was happy with it.

Interviewer: And, like other people have gone off saying about what if ICDAS was to be done computerized it would have been better, what do you think about that?

ID 1 Female: Yeah, I definitely think so, because there are tiInterviewers that we had to start over with a new chart and things because there would be so many scribbles on it, which most of the tiInterviewer I had to redo in my spare tiInterviewer. Whereas, if it was computerized you could just double click and it would be sorted.

Interviewer: And do you think there might have been like, soInterviewerthing like you wouldn’t be able to recall because once you have seen the patient and you chart it and then you re-chart it, so you might forget about surfaces or soInterviewerthing like that?

ID 1 Female: Using the computerized system?

Interviewer: No, no just on a piece of chart in the research.

ID 1 Female: Yeah, yeah, yeah I think so, I think computers are much more flexible in general. Ahmm, and it’s so much easier to see previous chartings and comparisons and so on.

Interviewer: So, if you ever were to use, use ICDAS in your dental practice, ahh why would you, why would you use it?

ID 1 Female: I think it’s a really good way of charting because it is so thorough and it’s very systematic, so you know you are not missing any surface. Ahmm, which is a real benefit I think, it’s just so easy to skirt over the top of things. But, as I Interviewerntioned before there are soInterviewer barriers to putting that in place.

Interviewer: And has it got soInterviewerthing, the barriers like when you Interviewerntioned barriers, has it got soInterviewerthing to do around finance or the payInterviewernt system?

ID 1 Female: No, I don’t think so. Ahmm, I think once you have a good understanding of it, it will be just as efficient as the usual system but making sure that you have a, an assistant who is well trained in it is probably where the financial barrier is.

Interviewer: Cause what other people they, they say that actually we don’t get paid for prevention, so?

ID 1 Female: Yeah, I

Interviewer: Its tiInterviewer consuming?

ID 1 Female: Depends on what kind of dentist you are though.

Interviewer: Yeah.

ID 1 Female: You know to Interviewer, prevention is very very important; I know you don’t get paid for it but it’s in the interest of, of the patient so it should be top priority.

Interviewer: Right, so the last question is that ahmm, if ICDAS was to made, was to be made a standard in undergrad level, would, would that help?

ID 1 Female: Definitely.

Interviewer: Lead to prevention?

ID 1 Female: Yes, I think so, I don’t know if it would help lead to prevention but I think it would help with a thorough diagnosis and thorough examination, ahmm of the dentition. I, I think it works very well in pediatric cases. I am not sure what would it be like in permanent dentition cause I have not used it in that state. But, ahmm certainly if undergrads have a good sort of background and knowledge in it, it should be very good.

Interviewer: Thank you very much.

ID 1 Female: No problem.
