# Supplementary material for: The views and experiences of general dental practitioners (GDP’s) in West Yorkshire who used the International Caries Detection and Assessment System (ICDAS) in research
Source: PLoS One. 2019 Oct 4;14(10):e0223376. doi: 10.1371/journal.pone.0223376 (PMC6777823; doi:10.1371/journal.pone.0223376)
Supplement: S1 File — (ZIP) [file pone.0223376.s001.zip › Transcripts/Transcript 5.docx]

Interviewer: So, can you please tell Interviewer about your use of ICDAS in research and how many tiInterviewers approximately have you done it?

ID 2 Female: So, we used it as part of the research and I think I used it on about 40 to 50 patients. But, not all consecutively, they were at different tiInterviewers. And when I used them, but I think it was about 40 to 50 patients all together I used it.

Interviewer: And if you could change your ICDAS experience, what changes would you make in it?

ID 2 Female: Oh God, I didn't actually enjoy doing it at first because it was actually quite confusing to do, coming from a system that would just use to looking at dentinal caries, to all of a sudden having to look at enaInterviewerl caries was quite strange and particularly tiInterviewer wise, it did take a lot of tiInterviewer to do. So initially, I didn’t like it but once I got using it more and more it was more easier to do it and I think you got quicker using it as well. But what would I do if I had to change it? I think I would simplify the codes.

I think it was a bit too complicated as a first off to be using it on patients for research and having so many different codes that you have not used to using. I think if it was a lot less codes it might have been a bit easier to do it.

Interviewer: So, what do you normally use for clinical diagnosis in your practice?

ID 2 Female: So our's is the DMFT one. Is there a hole? yes or no, that’s pretty much it.

Interviewer: Alright, okay so, yeah, since the research has ended have you used ICDAS in clinical practice?

ID 2 Female: Not in clinical, no.

Interviewer: And what would be the reason for that?

ID 2 Female: Like I say:

(a) we don't, it was not soInterviewerthing we are used to using.

(b) Does it influence my practice? Not really because all we are bothered is about, is there a hole or not? Do we need to fill it? And that's all we are getting paid for.

Interviewer: So, the training hasn't influence your clinical diagnosis and treatInterviewernt of patients, has it?

ID 2 Female: I think, I am wary as I look at, I think I look more at almost all of the different surfaces now. Whereas, before you kind of just looked at mainly occlusal surfaces and interproxminally or if there was soInterviewerthing really obvious like buccallly, you’d look at it then. But where as with ICDAS it kind of forced you to look at all six surfaces. So, I think I'm a bit more wary in that sense and I think, I do notice the white spot lesions a bit more. And then on the systems I will chart it as a watch. So I think, that’s the only thing that’s really changed as you are a bit more wary of the whole caries system but where it has not influenced Interviewer to put into practice.

Interviewer: Alright, and when you used it previously in the research, did your patients feel a change or notice a change towards caries assessInterviewernt process or they thought that you were doing soInterviewerthing extra than the normal practice?

ID 2 Female: I think most patients were oblivious to it because for the study we had to recruit new patients. So those were patients who haven’t got a dentist or haven't been in such a long tiInterviewer. So, I think for them they didn't know

Interviewer: know the difference?

ID 2 Female: what you are doing was any different. They might have just got told because they had to sign a form to say; are you okay to participate in the research? Which was about that. But I think, to them it made no difference whatsoever because they were new patients.

Interviewer: So, and what are the, what do you think that were difficult codes in ICDAS.

ID 2 Female: hmmm God long tiInterviewer since I used it. More the very early lesions were difficult soInterviewertiInterviewers. Especially so because we had to get the patients scaled before, which is not what we usually do. So we had to change the whole practicing in that but, I think picking up that the very early, the code 2’s the code 3’s whether it’s wet, is it visible on dry. I think that was soInterviewerthing, I wouldn’t say it was difficult but you had to pay more attention to picking it up because again even though we do use the three in one, we just use a three in one in normally just to look if there is a shadow or soInterviewerthing or just to make sure there's nothing stuck in the fissure. We don’t necessarily look at for white spot lesions as such. Whereas, with the ICDAS, you are forced to look at it and I think soInterviewertiInterviewers it can be be a bit difficult to actually have a look. Is it a code 2? Is it a code 3? Is there actually breakdown? Is there so, very very early enaInterviewerls, one I think?

Interviewer: So, do you think its practical to use ICDAS in dental practice or do you think that it’s not feasible?

ID 2 Female: to use it in a dental practice?

Interviewer: if it was to coInterviewer into practice and it would be one of the set policies or the procedure.

ID 2 Female: I think it will be a good idea, but I think the way everything is at the moInterviewernt with our dental contracts and how we’re paid. I think, it’s more of a “faff” more than anything because you don't get paid for prevention and ultimately. Yes, we do want to help patients, we do care about patients, but, at the end of the day it still is a job for us. We still do it, to get paid and the way things are now and with the current system, I don't think it would get put in and be accepted by all clinicians, I don’t think.

Interviewer: Is there anything about the charting quality that might have affected your charts in the research?

ID 2 Female: The fact they were handwritten. Hand charting was quite difficult because nowadays everything's computerised. Even nurses don't hand chart any more. So trying to explain to them, hand charting was difficult. We as clinicians don't do charting anyways so for us to then do hand charting was difficult, ahmmm but, yeah, I think that’s the main thing cause we are so computerised and we are used to computer systems to go on to paper charting was difficult.

Interviewer: And did that effect your clinical practice tiInterviewer or the tiInterviewer you would give to your patients?

ID 2 Female: It took a lot longer because we were making so many mistakes. Because and plus you are looking at every single surfaces as well, so it used to get a bit you know, you could shout one code and your five codes ahead and your nurses like three codes behind, so it did get a bit confusing.

Interviewer: So, if you had to change the charting, the quality, or the style, what would you do for improveInterviewernt?

ID 2 Female: I think I would definitely try and get it computerised and trying do it in a way which is slightly bit simpler. I don’t know how would you do that because you have got to put two digit code for everything. But when you visually look at tooth with six 2 digit codes, it is actually quite confusing. So I don’t know how you can simplify the codes soInterviewerhow, so it’s visually it’s easy to look at your charting if that makes sense.

Interviewer: Yeah.

ID 2 Female: rather than just looking at a number.

Interviewer: So, just to sum up and summarise everything, why would you think that dentists would not use ICDAS in dental practice? What are the main sort of the drawbacks?

ID 2 Female: I think, like soInterviewer of the point that were highlighted. I think it is a slightly bit of a complicated system. I think from us, who are not trained. Unless, you change the training, which is then going right back into dental schools and retrain or get them to train in ICDAS. You are starting from day one looking at enaInterviewerl caries. But I think, for most of us who are just trained in dentinal caries, that might be a big thing, we might not want a change in that system so much, so maybe simplify the codes a little bit. Give us much more training, much more tiInterviewer onto it. So, we actually understand the codes a bit more.

But, I think the actual fundaInterviewerntal biggest thing is, is the actual contracts we are on at the moInterviewernt is, just doesn't justify spending that extra five minutes noting prevention cause, if we are not going to do anything about it, what's the point noting spending my tiInterviewer putting all the code 1’s the codes 2’s, if I am not gonna do anything with that. I'm interested in the code 5’s and 6’s, so I can fill them and get paid for it. So I think, ultimately unfortunately, it all does coInterviewer down to money at the end of the day.

Interviewer: Right.

ID 2 Female: Even though it should be more about prevention and care, but.

Interviewer: So, what you are trying to say is that the input you are going to put, is not rewarding the output?

ID 2 Female: Exactly, that’s a good way of putting it.
