# Supplementary material for: The views and experiences of general dental practitioners (GDP’s) in West Yorkshire who used the International Caries Detection and Assessment System (ICDAS) in research
Source: PLoS One. 2019 Oct 4;14(10):e0223376. doi: 10.1371/journal.pone.0223376 (PMC6777823; doi:10.1371/journal.pone.0223376)
Supplement: S1 File — (ZIP) [file pone.0223376.s001.zip › Transcripts/Transcript 6.docx]

Interviewer: Hello I'd like to interview you about the, your ICDAS experience. So my first question would be, tell Interviewer about, how did learn ICDAS and how many tiInterviewers have you approximately done it?

ID 3 Female: Well, I learn it through e-learning, it was 90 minutes online course. And, how often I used it? We used it about a month. I would say, I used it randomly, not on every patient but for a month.

Interviewer: Right, and if you could change your ICDAS experience, what changes would you make?

ID 3 Female: Ahmmm, what I would say initially, I found it a bit difficult. But, then I get used to it and now I would like to go for more advanced and erm, more extensive course and really, I would like to learn more about this system.

Interviewer: Right, and has the training influenced your clinical diagnosis and treatInterviewernt of patients?

ID 3 Female: Yes, I will say yes, because with the use of this system. I'm just now looking more forward more towards prevention, rather than just treating caries. So, actually I am looking more towards the cause and just preventing it.

Interviewer: Right, so what system do you normally use in the dental practice to detect caries?

ID 3 Female: In our dental practice we used to use the DMFT standard, because it's very quick and it’s easy as well.

Interviewer: And, how often do you use the the system in your dental practice and was there a culture shift from your normal caries diagnosis practice and using ICDAS?

ID 3 Female: Ahmmm, yes the DMFT system we used it very regularly, almost on each and every patient. And, yes there was a cultural shift and I had to discontinue ICDAS because my patients were taking longer to deal with and because of the works strain, the rest of the waiting list was just getting impatient. So, I had to discontinue it.

Interviewer: And, how did the dental nurses react or feel, or did they not notice a change in the caries assessInterviewernt process?

ID 3 Female: The dental nurses. No, they did note a change and to to be honest they really appreciated it. But, they were not very happy with the record keeping, because I think so they found it quite tiInterviewer-consuming.

Interviewer: Right, and so why wouldn't you use ICDAS in dental practice?

ID 3 Female: I would say, we as a dentist are not paid for prevention and by the end of the day clinical decision-making are influenced by other factors as well.

Interviewer: And so tell Interviewer about the difficult codes in ICDAS? What did you personally find difficult?

ID 3 Female: Personally, hmmmm I think I found code 3 and code 4 as well. Because, there was underlying dark shadow of dentine. So, I found these codes quite difficult.

Interviewer: And, tell Interviewer about the charting quality, is there anything, which may have affected the quality of your charts.

ID 3 Female: Because, it's all on the paper so the charting was a bit difficult to deal with. And, I think so with the new technology, even the ICDAS should be computerised.

Interviewer: Alright, thank you very much.

ID 3 Female: thank you, thanks.
