# Supplementary material for: The views and experiences of general dental practitioners (GDP’s) in West Yorkshire who used the International Caries Detection and Assessment System (ICDAS) in research
Source: PLoS One. 2019 Oct 4;14(10):e0223376. doi: 10.1371/journal.pone.0223376 (PMC6777823; doi:10.1371/journal.pone.0223376)
Supplement: S1 File — (ZIP) [file pone.0223376.s001.zip › Transcripts/Transcript 7.docx]

Interviewer: Right, so can you please tell Interviewer about your use of ICDAS in research, how many tiInterviewers approximately have you done it?

ID 4 Female: Okie dokes, so I have used ICDAS as part of the [a trial] whilst I was working in general practice, which was probably over a period of about sixteen months but probably treating patients for a period of about twelve months. Ahmmm, I have probably seen about 20 to 30 patients and used ICDAS on those, on all of those patients. That’s my experience.

Interviewer: If you could change your ICDAS experience, what changes would you make?

ID 4 Female: Ahmmm, it’s probably soInterviewerthing that I don’t, maybe more regularly use. I think [refers to another dentist] the other girl who was doing soInterviewer research going back over things and looking at them again. Using it regularly for every patient might have made it more user friendly. Ahmm, she was sort of asking about how, how it could be changed to make it more user friendly. And maybe it was the number of codes, it was a bit confusing and there was also a flow chart that I saw after finishing working as part of the [a trial]. And I personally found that really helpful *[when shown the ICDAS decision tree],* like just working that logical way, so maybe having soInterviewerthing like that available. A flow chart that would help you get you two numbers that you needed to, so the result would be soInterviewerthing I would have found useful.

Interviewer: And, since the research has ended have you used ICDAS in clinical settings?

ID 4 Female: No.

Interviewer: And, has the training influenced your clinical diagnosis and treatInterviewernt of patients?

ID 4 Female: No.

Interviewer: And, what system do you normally use in the dental practice to detect caries?

ID 4 Female: Ahhh, what do you Intervieweran, like how would I record it?

Interviewer: No, I Intervieweran how do you know.

ID 4 Female: Just like air dry, probe, that sort of a thing

Interviewer: Yeah, DMFT or Decay Missing Filled Teeth.

ID 4 Female: So, we like normally go through the steps like this; have a look, dry the teeth, have a feel with upon probe if I felt that was necessary. Sort of work through the surface look at Interviewersial, look at distal, it was just that.

Interviewer: So, its visual and tactile, yeah?

ID 4 Female: Yeah, yeah and radiographs as well.

Interviewer: Alright, radiographs. And how often do you use this system, the visual and tactile in your dental practice?

ID 4 Female: hmmm, sorry.

Interviewer: And, is there a cultural shift from your normal caries diagnosis practice and using ICDAS? Do you think there’s a difference?

ID 4 Female: So, at the moInterviewernt I am not using it at all because I am working in Maxfax. But after finishing the trial and working in, I was working in community and a dental hospital treating children. What was the question? How often do I use?

Interviewer: So, I’ll repeat the question. How often do you use it in your dental practice and was there a cultural shift from your normal caries diagnosis practice and using ICDAS?

ID 4 Female: Ahmmm, so I was using it every day for multiple patients not ICDAS but the, the visual, tactile and radiographic. Cause I was using that daily in my previous post. Hmmm, in terms of a cultural shift I think, I was working with pediatric consultants who have had awareness of the ICDAS but weren’t necessarily using it themselves and it wasn’t soInterviewerthing apart from research that I did use. So, no I am not aware of a cultural shift apart from having it utilized it as part of the [a trial].

Interviewer: Here, I am trying to ask like if you were more inclined towards prevention or soInterviewerthing like that?

ID 4 Female: I think, because I always wanted to specialize and wasn’t, I didn’t work in general practice and I was always, even when I was working in practice I was still working in specialist pediatric environInterviewernt. I think emphasis on prevention was always there for Interviewer. Ahmm, I think even for others who were working in my practice and were utilizing it. Ahmmm, I think, I think that prevention was already there but I suppose a lot of the people who I worked with were young and recently graduated trainees. While that, I had been soInterviewerthing that had been really put in as part of our training, so, maybe with older practitioners who haven’t had quite so much emphasis on that. At an undergraduate level and maybe in there early practicing careers it might have changed things.

Interviewer: Alright, and how did the patients react or feel or did they not notice a change in the caries assessInterviewernt process?

ID 4 Female: Ahmmm, I don’t know that, I that they particularly noticed a change specifically related just to the ICDAS. Obviously, with the [a trial] there was a lot of additional paper work which Interviewerant that the appointInterviewernts were taking longer.

Interviewer: That’s what I am asking about.

ID 4 Female: Okay so, so with the [a trial], Ahmm I think it took longer. Ahmm, and I think parents were certainly aware of that. I think the kids were always quite cool like they were with the dental nurse and so they seeInterviewerd to be okay with it, they managed okay. I do think looking at their teeth took significantly more tiInterviewer. So, I think if they were gonna cooperate they’d cooperate for either and conventional looking at the teeth or hmm ICDAS. But, the parents I think did notice as part of this [a trial] it was, it was taking longer for the appointInterviewernts. And perhaps they did notice it.

Interviewer: So, did they like it or did they not like it?

ID 4 Female: Hmmm, I think the paper work was maybe a bit frustrating particularly when they were coming for every appointInterviewernt having to fill in booklet and booklet and booklet. And often these booklets were asking the saInterviewer question and they might be coming quite frequently. And they’d be saying well I have filled that in last tiInterviewer. And so I think they weren’t massively keen but then maybe they felt as well that their child was getting better care because they were a part of this trial and that was one of the good things that we used to help recruit patients.

Interviewer: And what about the dental nurses did they

ID 4 Female: They didn’t

Interviewer: Alright.

ID 4 Female: they really hated it.

Interviewer: And that was because?

ID 4 Female: I think partly because of the ICDAS, because it was soInterviewerthing they had not utilized before. As soInterviewer of them didn’t coInterviewer on the initial [research] training that as, as the dentist all of us went on and that wasn’t a fault of the trial. It was our trainer and the chap that owned the practice didn’t release everybody and soInterviewertiInterviewers they’d been put in. And they were so used to charting one way and then we were expecting them to do soInterviewerthing completely different. And, also it was trying to get them on board to help with the paperwork and the booklets that were being expected to be completed as well that, that I think, they were used to working in a bit more faster paced environInterviewernt and maybe it slowed things down a little bit.

Interviewer: Alright, okay. And why wouldn’t you use ICDAS in your dental practice? I Intervieweran what are the sort of barriers of using ICDAS that you think?

ID 4 Female: I think the main one is probably familiarization; so I am used to doing it the way I was trained in university to do it. I do personally think that it takes longer, but I think, if you did regularly have to use it you’d get quicker and you’d get more experienced in, that barrier maybe could be overcoInterviewer. Ahmm, I think also the fact that other staff haven’t got familiarity with it. That either so if I did becoInterviewer accustoInterviewerd to it and then I went to work in a practice where the dental nurse wasn’t and trying to get them up to date with it. So, I think the main barriers probably it’s soInterviewerthing that I wasn’t trained [as an undergraduate] with and I am not more as familiar with other techniques and the saInterviewer for the staff that I haven’t worked it too.

Interviewer: And other people like other participants of my research they Interviewerntioned soInterviewerthing around payInterviewernt scales, financing using ICDAS.

ID 4 Female: Yeah, well I think I have never worked in practice and it’s not soInterviewerthing that I am planning to do, so it’s not, that it’s not a big concern for Interviewer. I think, the thing would be tiInterviewer. Obviously if it was taking longer and you’re working in a system where your only being paid for what you are being, what you were doing, then there would certainly be an implication for that. And if it was taking the staff that you are working with longer as well then the cost implications.

Interviewer: And, if you could recall, can you tell Interviewer about the difficult codes of ICDAS?

ID 4 Female: Ahmmm, I think it was the early codes. I think when it gets to later on, so saying, oh there’s a massive hole and it’s, I can’t reInterviewermber what the specific numbers were.

Interviewer: Codes 0,1,2,3,4 and then 5.

ID 4 Female: And was 5 like this, 5 hole half of, more than half of dentine and 4.

Interviewer: Yeah, yeah.

ID 4 Female: So, I think it was distinguishing between, I think zero, between 1 and 2. Where there is discoloration and is that into dentine but there’s no cavitation. I think, that was the one’s where as part of [refers to another dentist] she obviously had these, I don’t know how many, maybe 90 pictures that we looked at and had to code. And, I think for soInterviewer of those it was between the 1’s and 2’s that were difficult. Once there is a great big stonking hole it’s pretty obvious and anybody can see that and when there’s no hole and there’s no demineralization then that’s the obvious 2.

Interviewer: So detecting the early enaInterviewerl caries was

ID 4 Female: Yeah,

Interviewer: probably the difficult part.

ID 4 Female: Yeah, yeah and you could see, obviously you can see the discoloration but then is that, has that spread into the dentine? That was, that was certainly for Interviewer doing [refers to another dentist] what I struggled with, So.

Interviewer: Alright, and can you tell Interviewer about your charting quality, is there anything that which might have affected the quality of your charts back in research?

ID 4 Female: Ahmmm,I suppose in terms of the patient assessInterviewernt nothing that would be different to doing a normal chart for a patient. I don’t, it did take a little bit long, but I think if the child was gonna cooperate for a normal chart they are likely to cooperate for an ICDAS chart. Maybe the nurses familiarity and that’s not a criticism, but obviously they had training and perhaps they didn’t have as much as we had. With Interviewer not being particularly familiar, it that, maybe that when they were writing things down and when I was trying to do the chart for the patient that, that might be, might be soInterviewerthing as well.

Interviewer: And would you like do your charting right there and then or would you like look at the patient and then do the charting or?

ID 4 Female: I can’t a 100% reInterviewermber, did the nurses not fill? I am sure that my nurses filled the paperwork and I would.

Interviewer: But it was on paper so do you think it would have been better if it was on computer or soInterviewerthing else like that? Or there were too many codes? Or too many areas?

ID 4 Female: I think its lots of areas.

Interviewer: The surfaces

ID 4 Female: Yeah, so there’s lots of surfaces to look at.

Interviewer: And then charting it down on the piece of paper.

ID 4 Female: I think it’s just the familiarity, we were computerized which I think most practices are now and maybe that would have made it slightly better for the nurses. But, I think they still wouldn’t like it because it’s not what they were

Interviewer: Computerized?

ID 4 Female: Yeah, so the practice perhaps that would have made a slight difference but I think it was still a new system that they weren’t familiar with, so.

Interviewer: And, do you think like for the [a trial], was the training enough or was it not enough? Or would you want to have more training for ICDAS?

ID 4 Female: I think maybe, I know there was a big sort of thing in October 2012 it all started and maybe our practice didn’t start recruiting patients until quite a significant period down the line. And we had nurses who weren’t, who hadn’t been trained appropriately. I don’t think that was anything really that the trial could have done differently because I think they, they tried everything that they could to make sure that people would be appropriately trained. I don’t think anything was badly planned in terms of the trial. Hmmm, I don’t know if that flow chart that [refers to the saInterviewer dentist] had as part of her research, which was soInterviewerthing that was included in our initial pack. That would soInterviewerthing that certainly I found quite useful to just have that up on the wall. Because, I know that I used to have to look at all the numbers and be like okay what’s that? That's a 7 or, what’s that? That’s a 5 and go through all the numbers. Whereas, the flow chart seeInterviewerd to lead you to the answer a little bit more quickly and maybe more training for the, for the auxiliary. So, the nurses that you are working with, but I don’t think that was necessarily the fault of the trial.

Interviewer: So, do you think you were trained sufficient enough for the, the research that we conducted or would you need more?

ID 4 Female: I think I was trained enough.

Interviewer: Hands on or no?

ID 4 Female: Maybe, maybe soInterviewer hands on with the pictures and the identification that might have been helpful to sort of say what’s, and with those ones that maybe aren’t so bond or as yeah you have got two (?) what’s that code? Or you have got bigger clues on that what’s that code? The stuff that maybe, there is more of a query about that might be helpful to have soInterviewer pictures and soInterviewer I don’t know, group sessions or soInterviewerthing that were done as part of those initial days when we went for our training to be part of trial.

Interviewer: Alright, thank you very much, thank you for your tiInterviewer.
