# Supplementary material for: The views and experiences of general dental practitioners (GDP’s) in West Yorkshire who used the International Caries Detection and Assessment System (ICDAS) in research
Source: PLoS One. 2019 Oct 4;14(10):e0223376. doi: 10.1371/journal.pone.0223376 (PMC6777823; doi:10.1371/journal.pone.0223376)
Supplement: S1 File — (ZIP) [file pone.0223376.s001.zip › Transcripts/Transcript 8.docx]

Interviewer:Ahmmm, can you please tell Interviewer about your use of ICDAS in research and how many tiInterviewers approximately have you done it?

ID 4 Male: I used it for the, well, the [refers to a university trial] that we did. I was vaguely aware of it before, hadn't used it, I haven't used it since. Ahmm, so yeah, just for the number of patients which was sought for the [university trial].

Interviewer: And how many were they?

ID 4 Male: I would guess maybe about 30 or 40 patients roughly, around about that.

Interviewer: If you could change your ICDAS experience, what changes would you make?

ID 4 Male: I didn’t find it anyway useful. Ahmmm, so I probably just wouldn’t use it.

Interviewer: You wouldn’t use it?

ID 4 Male: No.

Interviewer: Alright, and since the research has ended have you used ICDAS system in clinical practice?

ID 4 Male: No.

Interviewer: Right, has the training influenced your clinical diagnosis and treatInterviewernt of patients?

ID 4 Male: No.

Interviewer: What system do you normally use in the dental practice to detect caries?

ID 4 Male: Ahmmmm, seeing, visualising things, drying them, using a probe, radiographic, ahmm and taking bitewings. That’s mainly it.

Interviewer: So, do you use DMFT or tactile visual?

ID 4 Male: No, oh well tactile visual yeah but not DMF.

Interviewer: So, more like treatInterviewernt based?

ID 4 Male: Yeah.

Interviewer: And, how often do you use the system like the treatInterviewernt based system that you using in your dental practice and is there a cultural shift from your normal caries diagnosis practice and using ICDAS?

ID 4 Male: I would use the, well the normal way I would go about diagnosing caries with every patient every day. So, it depends on how many people I see, 20-30, and 40 people a day, every day. Ahmmmm, with ICDAS I haven’t really incorporated it in with what I do. So, I wouldn’t tend to use it, at all. I don’t think it’s changed what I do, very much.

Interviewer: Why wouldn’t you use ICDAS in your dental practice?

ID 4 Male: I found it very complicated, I found it unnecessarily. So, I didn’t find it provided a great deal of benefit to diagnose in the caries or treating it. Ahmmmm, I might be quiet old school but I tend to see it very simply. If there is caries, is it soInterviewerthing that is monitor-able? Or can it be treated with fluoride to try and prevent it? In my experience largely, if there is carious lesion, there is going to be more of a carious lesion the next tiInterviewer you check it. Regardless of patient OH and fluoride applications, largely compliance even though you may try to educate them as much as you can, it isn’t high on a patient’s agenda. And that’s unfortunately, how it is in the real world. So, university teaching ideally, yes I know, hit them with fluoride to trying to reduce the lesion. In reality I tend to find that if you think it needs filling, you may as well fill it because there’s going to be a bigger hole next tiInterviewer.

Interviewer: How did the patients react or feel or did they not notice a change in the caries assessInterviewernt process in the research.

ID 4 Male: They didn't know. I Intervieweran it took longer to do the checkup but ahhh the way we did it. Most of them were new patients so they didn’t really have a great deal, they didn’t have anything previous to base that on. So, relatively yeah, they didn’t really appreciate the fact that, they knew they were a part of the study but in terms of the assessInterviewernt process, not really.

Interviewer: And, how did the dental nurses react or feel, or did they not notice a change in the caries assessInterviewernt process?

ID 4 Male: Ahmmmm, again everything took longer, which that's largely the sort of biggest factor. Ahmmm so, apart from that, no, probably not a huge amount of influence.

Interviewer: So, were they in favor or were they not.

ID 4 Male: If it took longer, no.

Interviewer: Alright, can you tell Interviewer about the difficult codes in ICDAS.

ID 4 Male: Ahmmm, the, just the complexity of ahhh categorizing all the lesions. Also, the recording system. I suppose the more you do it the easier it gets cause you learn through the repetition. But, as I said it’s largely, I felt unnecessary to make things that complicated.

Interviewer: And can you tell Interviewer about the charting quality is there anything which may have affected the quality of your charts?

ID 4 Male: The complicated system and ahhh although the instructions were quiet clear in trying to categories each lesion. I think, there probably was maybe a range of interpretations across the dentists that would use it, as to which it would fall into. So, that would probably, you know make the results of the study less viable. I suppose.

Interviewer: And, coming back to the why wouldn’t ICDAS be not feasible in part of dental practice, do you think it has soInterviewerthing to do with like e.g. finance, or?

ID 4 Male: Well, Yes, because I Intervieweran in an ideal world it wouldn’t matter how long things took, what things cost. But in the normal world of NHS dentistry you have a limited amount of tiInterviewer for people. Unfortunately, that’s the way it is. So, soInterviewerthing that takes longer for no conceivable benefit, isn’t gonna go done very well or be used very much.

Interviewer: So, it’s more about prevention than treatInterviewernt?

ID 4 Male: Well, yeah I Intervieweran as I said, its, I would still do the saInterviewer prevention, I would always have done. Ahmmm, it’s largely the treatInterviewernt of the lesion that is the caries is unaffected, it’s the system I didn’t find was providing any useful change to work in practice.

Interviewer: And, was it like tiInterviewer-consuming?

ID 4 Male: Yeah.

Interviewer: Yeah, so it would be hard to bring it into a clinical setting?

ID 4 Male: I think so, ahmmm certainly an NHS clinical setting. It’s, I feel probably unnecessary so largely that’s the way I would tend to make most of my practicing decision. If it’s not going to benefit the patient or myself, in terms of making my job easier or making the treatInterviewernt easier and it’s gonna take longer. Largely that’s not really gonna work.

Interviewer: So, in the NHS setting, I Intervieweran what influences your caries decision making, is it the payInterviewernt system or is it the amount of patients that you see or what is it?

ID 4 Male: Ahmmm, I suppose the patients themselves, they would influence it in, if you can see a relatively smaller lesion but you know that regardless of your instructions their OH is not up to scratch or their diet, they’d still have the cariogenic diet. Leaving a smaller lesion just probably isn’t gonna gain you anything other than a bigger cavity next tiInterviewer. Ahmmm, I have been at this practice for quite soInterviewer tiInterviewer. So, most of my patients are reasonably stable. The number of high caries patients, higher caries risk patients I see is quite small, but ahhh most of the tiInterviewer, it’s basically if I have decided soInterviewerthing is carious or going to becoInterviewer carious then that's the main sort of decision that we are making whether to restore.

Interviewer: And if ICDAS was to be taught in undergraduate level, do you think that this thing could be leading soInterviewerwhere?

ID 4 Male: Yes.

Interviewer: Like previously you Interviewerntioned you were old school.

ID 4 Male: Yes, yes.

Interviewer: Like whatever, you were taught in school is you are just doing that in clinical practice?

ID 4 Male: No, because things that we are taught in dental school are fine and it’s good to have the gold standard and the best way of doing things. Whenever you get into in a general practice, you tend to go with what you find works and what’s, that’s not what always you get taught in practice, ahhh sorry, what you get taught in university. It’s not always what works in practice.

Interviewer: So, practically it’s different than theoretical learning?

ID 4 Male: Yeah, absolutely.

Interviewer: Alright then, Thank you very much, thanks you for your tiInterviewer.

ID 4 Male: No problem.
